# Cryotherapy for Primary Treatment of Prostate Cancer: Intermediate Term Results of a Prospective Study from a Single Institution

**DOI:** 10.1155/2014/571576

**Published:** 2014-02-20

**Authors:** S. Alvarez Rodríguez, F. Arias Fúnez, C. Bueno Bravo, R. Rodríguez-Patrón Rodríguez, E. Sanz Mayayo, V. Hevia Palacios, F. J. Burgos Revilla

**Affiliations:** Urology Department, Ramón y Cajal Hospital, University of Alcalá de Henares, Colmenar km 9,100, 28034 Madrid, Spain

## Abstract

* Purpose.* Published data about cryotherapy for prostate cancer (PC) treatment are based on case series with a lack of clinical trials and the inexistence of a validated definition of biochemical failure. A prospective study with standardized followup protocol was conducted in our institution. *Material and Methods.* Prospective study of a series of cases including 108 patients diagnosed with localized PC at clinical stage T1c-T2c treated by primary cryoablation and median followup of 61 months. Criteria of biochemical recurrence were unified according to the American Society for Therapeutic Radiology and Oncology (ASTRO). End points were biochemical progression-free survival (BPFS), cancer-specific survival, and overall survival. Rate of complications was reported. *Results.* The BPFS for low-, medium-, and high-risk patients was 96.4%, 91.2%, and 62.2%, respectively. Cancer-specific survival was 98.1%. Overall survival reached 94.4%. Complications included incontinence in 5.6%, urinary tract obstruction in 1.9%, urethral sloughing in 5.6%, haematuria in 1.9%, perineal pain in 11.1%, and prostatorectal fistula in 0.9%. Erectile disfunction was found in 98.1%. *Conclusions.* Cryotherapy is an effective and minimally invasive treatment for primary PC in well-selected cases, with low surgical risk and good results in terms of BPFS, cancer-specific survival, and overall survival.

## 1. Introduction

The wide range of treatment options for clinically localized prostate cancer includes radical prostatectomy, radiation therapy (external beam radiation therapy (EBRT) and/or brachytherapy), or even more conservative approaches as active surveillance and watchful waiting [1].

Currently new technologies are being implemented with guaranteed limits of oncological efficacy and a clear benefit to the patient and the healthcare system. Since 1996, cryotherapy has been established by the American Urological Association (AUA) as a therapeutic option for the treatment of localized prostate cancer and in 1999 Medicare and Medicaid approved the cryosurgery as primary treatment of prostate cancer moving in the United States from the category of research to a clinical practice application recognizing cryotherapy as a therapeutic option. With short-term results, effective, safe and an acceptable adverse-effect profile has been proved; however, studies with longer followup [2] are needed. The existing data in the literature are based on case series and a few randomized studies comparing cryotherapy to the other standards of treatment. The criticism in published studies lies in the short followup of patients, the absence of unified criteria of biochemical recurrence and success and the absence of posttreatment protocolized followup.

We present a prospective study of a case series with a maximum followup of 132 months, and 50% of the cases exceeding 5 years of followup. 20 patients were followed longer than 10 years. Median protocolized followup were 61 months (range 10–132), with criteria of biochemical recurrence unified according to the American Society for Therapeutic Radiology and Oncology (ASTRO), was conducted in our institution.

*Historical Memory and Physical Principles.* The use of low temperatures for the treatment of tumours goes back to the 19th century (Arnott, 1851) [3] in the treatment of cervical and breast cancer. In the field of urology Gonder et al. [3, 4] used transurethral cryoprobes to freeze prostate tissue for treatment of benign prostate hypertrophy. It was not until 1968 when Soanes used cryotherapy for prostate cancer therapy. In 1974, the transperineal approach was described by Megalli et al. [5] using probes of nitrogen. During the decade of the 80s with the development of ultrasound, an emergent interest in the technique is seen. In 1988 Onik et al. [6] applied the real-time monitoring of the freezing process with transrectal ultrasound. The improvement of the cryoprobes, with the combination of the ultrasound control and temperature m onitoring both at the prostate and surrounding tissues, which allows urethral warming has prompted the third generation cryotherapy technique as we know it today [7].

Cryotherapy induces cellular damage by direct and indirect mechanisms immediately at the time of treatment and also deferred in time [8]. The ultimate goal of cryotherapy is cell death by necrosis and apoptosis. The main mechanism of injury is by coagulation necrosis. The effect of ice on cell membranes is an immediate disruption by a mechanical direct effect. Proteins are denatured by dehydration. The rapid congelation and the slow warming produce a thermal shock that damages lipoproteins and induces sudden changes in the osmotic pressure, pH, and osmolarity. Indirect and delayed effects are primarily due to ischemic changes affecting microcirculation producing vascular stasis in the thawed tissue, hypoxia, and thrombosis [9–11] which increases necrosis by hypoxia in tributary territories. The edge of the lethal area should reach −40°C as Tatsutani et al. demonstrated in studies in vivo with neoplasic prostate cells, being the temperature at the edge the ball of ice 0°C [12]. The keys to cell destruction depend on minimum temperature reached, speed of freezing, freeze time, and the interval between freeze-thaw cycles [13, 14].

It is possible that some cells can escape to the lethal action of the cold (because of microvascular disturbance or directly) but it is also possible that sublethal damage drives the cell into a process of programmed death. Apoptosis occurs between 6° and 10°C and activation time needs minutes. In the periphery of the ice ball the maintained temperatures of 0°C many minutes can induce the setting up of this mechanism and increase the number of dead cells.

## 2. Material and Methods

Prospective study of series of cases (108 patients) treated by primary cryoablation for prostate cancer at our center were stratified according to the Gleason Score and D'Amico risk group. The low-risk group was defined as patients with clinical stage ≤T2a, PSA level <10.0 ng/mL, and Gleason score ≤6. The moderate-risk group was defined as patients with stage T2b, PSA level between 10.0 and 20 ng/mL, or Gleason score 7. The high-risk group included men with stage ≥T2c, Gleason 8–10, or PSA ≥ 20 ng/mL. A total of 114 treatments were performed including cases where a second procedure was repeated.

Inclusion criteria comprised patients diagnosed with localized prostate cancer at clinical stage T1c-T2c and negative extension studies were carried out whether value of PSA at diagnosis was above 20 ng/mL, with the exception of two patients treated in clinical stage T3aN0 M0 as described later.

All cases were carried out following the same surgical protocol, performed by the same surgeon in more than 80% of cases. The technique is usually performed under regional anaesthesia. The patient is prepared with a broad-spectrum antibiotic prophylaxis and cleaning enema. The patient is positioned in dorsal lithotomy, facilitating a good exposure of the perineum and the handling of transrectal transducer (longitudinal biplane probe to 7.5 Hz).

In all cases we used the Stryker Cryo/44 coaxial system with cryoprobes of 2.4 mm of diameter, in number of six to eight depending on the prostatic volume. Equipment used argon (300 bars of pressure and temperature of −180°C) for the freeze cycle and helium for the heating cycle (200 bar pressure with exchange of temperature of −180°C to 40°C in 30 seconds). Temperature is monitored inside and outside the prostate. The thermal sensors are placed in apex, external sphincter, and left and right neurovascular bundles. Hydrodistention of the prostatorectal area is done by injecting saline solution with broad-spectrum antibiotic at Denonvilliers' space such as protection of the rectal wall (Onik Manoeuvre) [15]. Control cystoscopy is performed to ensure the indemnity of the urethra, which is protected by using a continuous flow system with a pump pressure of 4.5 bar, approved by the FDA, which circulates saline with methylene blue at 41°C and keeps adjacent tissues to a temperature of 38°C. Two complete freeze/thaw cycles are performed. Depending on the prostate volume or on prostates with a longitudinal diameter greater than 35 mm, a third cycle is needed that tends to associate a 10 mm distal displacement of the cryoprobes, in a maneuver called “pull back.” Hospital discharge occurs in 24 hours, maintaining the bladder catheter two weeks and ambulant treatment with anti-inflammatory and oral antibiotics.

Biochemical recurrence was defined according to the Phoenix criteria defined by the ASTRO as a rise in prostate-specific antigen (PSA) of nadir plus 2 ng/mL.

The followup has been carried out with labs analytics with PSA every 3 months during the first two years of followup; every 6 months to five years; and subsequently annually. Prostate biopsy is performed at 6, 12, and 24 months and at the fifth year of treatment. Biopsy confirming local recurrence is mandatory in the case of PSA elevation above the established as cut-off point of biochemical recurrence.

The analysis of histological samples was made by specialized and limited uropathologist.

Descriptive variables are analysed by mean, median, standard deviation, and 95% confidence interval (95% CI). Survival analysis was carried out with the Kaplan-Meier method (K-M) and Log-Rank test to compare two or more K-M curves (statistical dependence) and association between different risk groups, clinical stage, and Gleason at the biopsy.

## 3. Results

We discuss the results of 108 patients who underwent cryotherapy as a primary treatment for prostate cancer with a maximum followup of 132 months, with 50% of the cases exceeding 5 years of followup. Median was 61 months (range 10–132). The middle age at time of treatment was 72,05 years. Only a single case was lost during followup.

A descriptive analysis of patients and tumours characteristics and associated comorbidities, as well as the descriptive analysis of the treated tumours, is shown in Tables 1 and 2, respectively.

Two cycles were made in 91 cases (84.2%) and a third cycle was required in 17 patients (15.8%) because of high volume or length of the gland.

**Figure 1:**
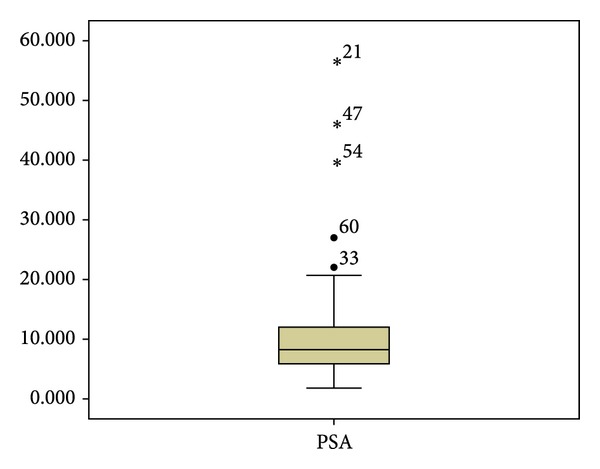
PSA distribution.

The median pretreatment PSA was 8.25 ng/mL with a confidence interval of 95% for the average of 9,013 to 12,021 ng/mL (Figure 1). 85% of the cases showed a Gleason ≤7 and half of the sample a Gleason ≤6. According to the clinical stages 52.8% were T1c and 57.4% of the patients were in the low and medium-risk categories.

**Figure 2:**
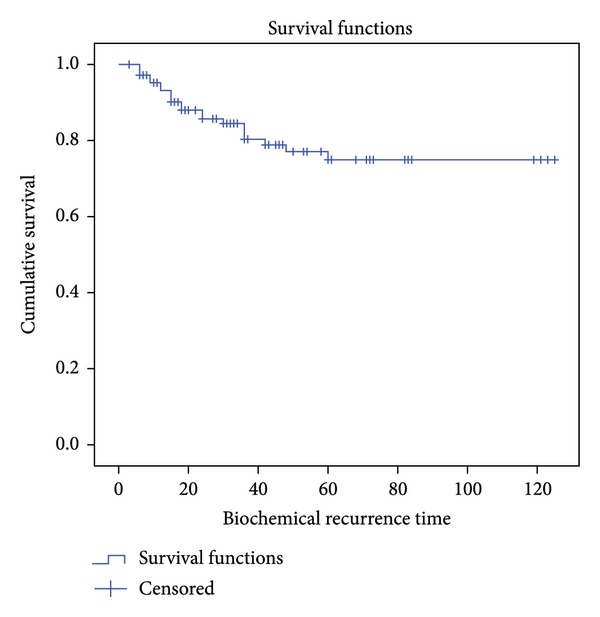
Global BPFS.

Biochemical relapse occurred in 21 cases and the definition of biochemical failure was accepted as an increase of PSA of Nadir plus 2 ng/mL according to previously established criteria. Applying the Kaplan-Meier curves, biochemical progression-free survival (BPFS) was 80.4% with an average of 100.2 months (90.9–109,6 months with 95% CI) as reflected in Figure 2.

**Figure 3:**
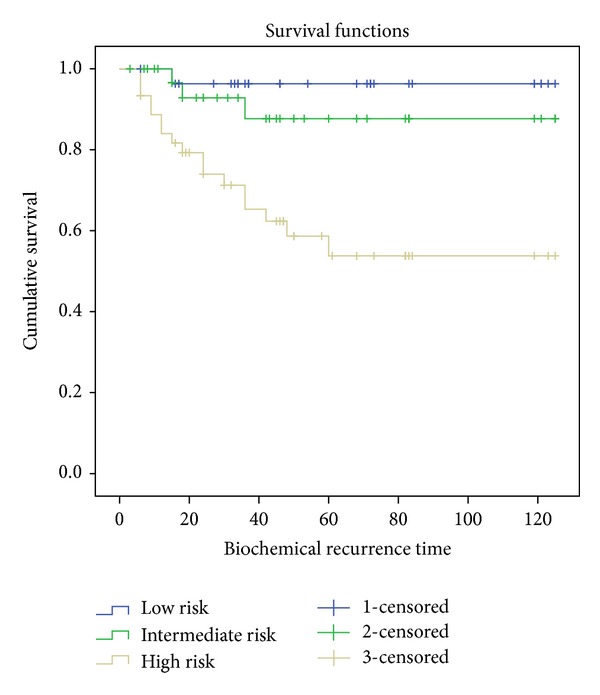
BPFS according to risk.

**Figure 4:**
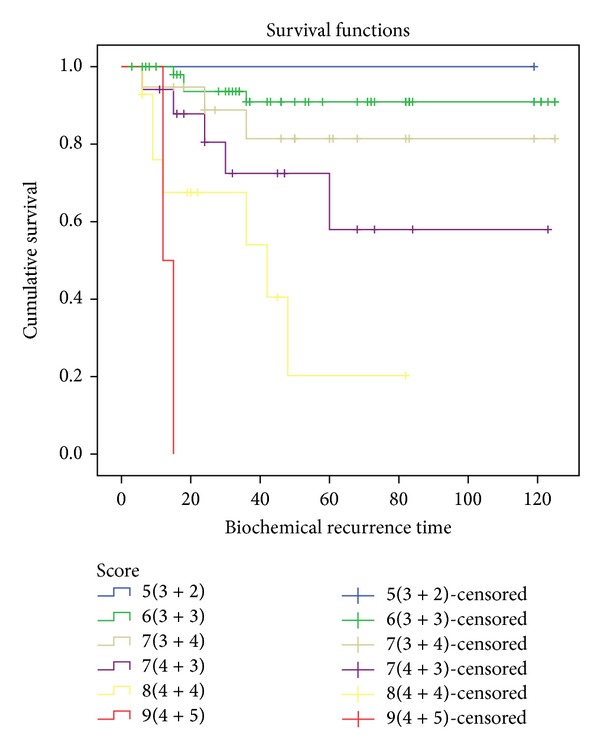
BPFS according to Gleason score.

Overall survival stratified by risk groups is reflected in Figure 3. The BPFS for low-, medium- and high-risk patients was 96.4%, 91.2%, and 62.2%, respectively. BPFS was 92.6% for Gleason 6 patients and the patient with Gleason 5 (3 + 2) has not shown evidence of biochemical recurrence. All cases with Gleason ≥7 (4 + 3) presented inferior BPFS time than the global BPFS for the total sample with values behind the 70% and less than 50% for patients with Gleason 8 and 9.  Figure 4 shows the survival curves stratified by Gleason score. Global BPFS data and 95% confidence intervals are presented in Table 3, stratified by risk and Gleason score.

Comparing survival curves with the Log-Rank test, statistically significant differences are only found in high-risk patients, maintaining a rate of freedom from biochemical relapse of 62% (*P* = 0.001 and *P* = 0.009), and not among low- and medium-risk patients for 96.4% and 91.2%. The Log-Rank test applied to the survival curves stratified by Gleason score only shows statistically significant differences for Gleason score ≥8 (4 + 4), but the small number of cases limits its interpretation (Table 3).

Followup protocolized biopsy at 6, 12, and 24 months and 5 years was only performed in the first 50 cases of the series. Results were repeatedly negative when PSA levels remained below the established level for biochemical recurrence. No cases presented positive biopsy findings in the absence of biochemical recurrence. However, due to the prospective nature of the study, 85 cases (78.7% of the sample) underwent protocolized biopsy as mentioned before with exactly the same results.

Among the 21 cases presenting biochemical relapse, a positive biopsy was detected in 7 (33%), including one patient with distal metastasis confirmed at bone scan. Conversely, in 5 patients (24%) biopsies were reported as posttreatment changes without signs of metastasic disease (bone scan and CT). Nine cases (43%) presented with imaging studies confirming the presence of metastasis or PSA doubling time making them suppose the existence of distant disease.

After recurrence diagnosis ten cases (47.8%) initiated treatment with androgen deprivation therapy (ADT) with a combination of bicalutamide and LHRH agonist, 5 (23.8%) remained under active surveillance, and 6 cases (28.4%) underwent a second round of cryotherapy. The recurrence and followup after salvage treatment is resumed in Table 4.

**Figure 5:**
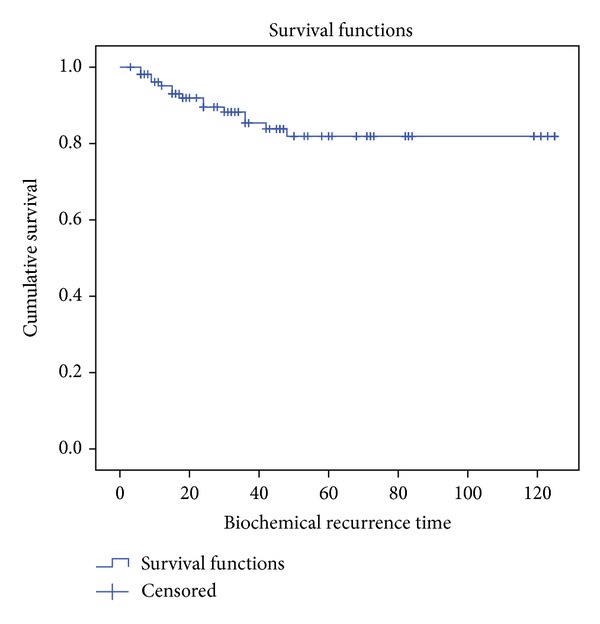
Global BPFS including salvage treatment.

Considering all treatments, the global BPFS was estimated in 88.1%, with a mean estimate of 106,862 months (typical error 4,298) and values for a 95% CI between 98,439 and 115,285 months. K-M survival curve is shown in Figure 5.

Rate of complications included incontinence in 5.6%. In our study we consider urinary incontinence according to the definition of the International Continence Society (ICS) (any involuntary loss of urine that is a social or hygienic problem) or requirement of ≥1 pad/day [16]. Urinary tract obstruction in 1.9% of the patients. Urethral sloughing occurred in 5.6%, haematuria in 1.9%, perineal pain in 11.1%, and prostato-rectal fistula in 0.9%. Overall impotence rate was reported in 98.1%, considering that 62% of these patients had erectile dysfunction prior to treatment. Erectile function was defined as an erection sufficient for unassisted sexual intercourse. Erection recovery with phosphodiesterase-5 inhibitor (PDE5i) was not achieved in any case. It is worth emphasizing that impotence was present in more than a half of the patients before treatment, and 35 patients were older than 75. Complication rates are shown in Table 5.

Exitus occurred in 6 occasions (5.6%). In 2 cases (1.9%) dead was related to prostate cancer (cancer specific) after biochemical recurrence. In four patients death resulted from causes unrelated to the disease. Cancer-specific survival was 98.1%. Overall survival reached 94.4%.

## 4. Discussion

Many studies have been published reporting the results of cryotherapy depending on patient and tumour characteristics, classified according to the extraprostatic progression risk and clinical stage. However, there is a lack of randomized studies and most of the publications are based on case series that include patients with any clinical stage, even locally advanced T3-T4. Studies also refer to treatments carried out with first generation devices that only managed five cryoprobes of bigger diameter and procedures that did not include the Onik maneuver for rectal protection, so the incidence of fistula incidence seems to be much higher than at present. Another fact is the definition of which PSA level should be used as cut-off point to determine biochemical failure, with no universally established consensus leading to a bias when studies are compared. Followup biopsies and a standard surveillance protocol are not standardized. Only Donnelly et al. in 2010 [17] communicate results of a randomized study comparing external beam radiation versus cryotherapy in patients with organ confined prostate cancer with a 7-year followup, but with a small number of patients in each branch (122 versus 122 divided into five groups according to clinical stage).

Our series is the continuance of a work begun in 2001 and carried out in collaboration with the Office of Evaluation of New Sanitary Technologies by Lain Entralgo Agency and sponsored by Carlos III Institute.

Nowadays, one of the problems in assessing the results of cryotherapy lies in the lack of consensus on the value of PSA used as cut-off point to define the biochemical relapse criteria. Levels of PSA of 0.1, 0.3, 0.4, and 0.5 ng/mL have been established as criteria of biochemical recurrence compared to radical prostatectomy. Given that cryotherapy is an interstitial procedure, it makes more sense a comparison with radiotherapy. This is the reason why the definition of biochemical recurrence of the American Society of Therapeutic Radiology and Oncology (ASTRO) seems more appropriate, using the Phoenix criteria (nadir of PSA +2 ng/mL) as a threshold to define the biochemical relapse.

Immediately after treatment serum PSA levels can arise because of intracellular PSA release by necrosis. The nadir value is generally reached after three months. Levels may not drop to undetectable levels by the persistence of viable periurethral prostate tissue [18].

In our series, routine determination of PSA was assessed every three months for 2 years; every 6 months up to 5 years and subsequently on a yearly basis indefinitely.

It has been shown that the low the PSA values achieved, the low the likelihood of positive biopsy results and elevation of PSA at followup. Control biopsies must be performed at least 6 months after the procedure to reduce the effect of inflammation on the gland. The indication of followup biopsy is not well established. Positive biopsy rates in the group biopsied based on suspicion of treatment failure due to increase in PSA were higher than in those in absence of biochemical recurrence (38.4% versus 15.4%) [19]. Elevated PSA values prior to treatment and clinical stage have been associated with positive biopsy results [20].

In our series protocolized biopsies are scheduled at 6, 12, and 24 months and 5 years, and eventually in case of sudden elevation of PSA levels. This scheme has been applied exclusively in the first 50 cases. Subsequently, and given the prospective nature of the study, biopsy has been done at 6, 12, and 24 months until the case number 85 (78.7%). Data obtained in our series were repeatedly negative when PSA values were kept below the established level for biochemical failure. Scientific evidence, the absence of positive findings for adenocarcinoma at biopsies in the absence of biochemical recurrence, and the risk derived from the realization of transrectal biopsy forced us to modify biopsy criteria. In fact, the only case of rectourethral fistula in our series, happened subsequent to the transrectal biopsy of 24 months, without signs of biochemical relapse and absence of malignancy in the samples sent to the pathologist. Up to this point the negative biopsy rate was 94.1% even in the presence of patients with biochemical recurrence. As described previously, 5 patients are kept under surveillance for presenting criteria of biochemical recurrence, negativity in prostate biopsy, and absence of distant disease, corroborated by a slow PSA kinetics and long PSA doubling time (>24 months). Currently, biopsies are only performed in case of PSA increase [18–22].

We believe that transrectal biopsy should only be done to confirm the existence of local recurrence once biochemical recurrence has been established.

According to the recommendations of the European Urological Association Guidelines (EAU 2012), potential candidates for cryosurgery would be patients at low risk of progression (PSA < 10 ng/mL, <T2a, or Gleason <6) or intermediate risk (PSA > 10 ng/mL or Gleason 7, or stage >T2b). Cryoablation of the prostate is recognized as minimally invasive, nonexperimental procedure, and a feasible option for treatment.

For the American Urological Association (AUA updated 2010) it is figured as an option in organ-confined disease, at any grade, showing absence of metastatic disease, and preferably at intermediate risk. It is also recommended prior lymphadenectomy or multimodal treatment if the risk of lymphatic involvement is greater than 25% according to established nomograms (i.e., Partin tables) by PSA > 20 ng/mL or Gleason 8–10.

Primary cryotherapy is a possible alternative treatment for prostate cancer. It is a recognized “option” accepted by AUA and EAU Guidelines (2013 Guidelines: Grade of Recommendation: C) for the treatment of localized prostate cancer. It could be indicated in patients at low risk of extracapsular disease but with high surgical risk, unfit for surgery or life expectancy less than ten years. Patients with a life expectancy longer than 10 years should be informed that minimal data are available on the long-term outcome for cancer control at 10 and 15 years. It should also be considered in patients who desire minimally invasive therapy for intermediate risk prostate cancer. However, patients who are bad candidates for surgery by associated comorbidity, obesity, previous pelvic surgery, or negative for signing of the informed consent, contraindications for radiotherapy (prior radiotherapy for rectal cancer, narrow pelvis, or inflammatory bowel disease) by their backgrounds may be candidates for treatment with cryosurgery, regardless of the risk, even assuming the possibility of a second treatment or combination therapies needed.

Comparison of treatment modalities from prostate cancer is complicated by the absence of uniform criteria to define results in terms of biochemical recurrence, the lack of randomized studies, being all available data retrospective, single centre reports, and also because of an inherent bias in patient selection. Another factor to bear in mind is that techniques and dose, especially in radiotherapy, have changed throughout periods of time and comparison between historical cohorts is difficult in this respect.

Compared to invasive treatments, note that patients who are candidates for ablative proceedings as cryotherapy are older than patients suitable for radical prostatectomy (RP). In our series median age was 72 years, being cohorts of surgery quite younger, around 63 years [23]. Only one study by Gould in 1999 [24, 25] compared cryotherapy with RP; it was a short series of patients and results were defined in terms of PSA after 6 months, achieving cryotherapy cohort at 0 PSA in 66.7% of cases compared to 48,2% in radical surgery group. Patients with PSA less than 10 were more likely to success. This study has several bias in terms of patient selection by the surgeon and small number of patients.

Active surveillance is an option in low-risk patients, with followup available data of less than two years. The largest cohort by Klotz et al. [26] with 450 patients with clinical stage T1c or T2a, PSA < 10 ng/mL were enrolled with an overall Gleason score <6 (PSA < 15), with patients >70 years having a Gleason score <7 (3 + 4). At a median followup of 6.8 years, the 10-year overall survival was 68%. At 10 years, the disease-specific survival was 97.2%, with 62% of men still alive on active surveillance. 30% of patients underwent a radical treatment; 48% for a PSA doubling time <3 years; and 27% for Gleason score progression, remaining 10% switch the treatment because of personal preference. Overall survival varies between series and time to followup from 70 to 100%. Biochemical failure after treatment in patients who underwent active treatment was 13% [27].

Most recent series of RP for low- and intermediate-risk prostate cancer (EUA guidelines) show 10-year PSA-free survival rates between 60 and 65% and 10-year cancer-specific survival of 94 to 97% with 53 to 153 months-followup. For high risk prostate cancer reported PSA failure rate remains in 44% and 53% at 5 and 10 years, respectively [28]. D'Amico et al. found a 50% risk of PSA failure at 5 years after RP [29]. Spahn et al. [30] published the largest multicentre surgical series to date, including 712, and reported a CSS of 90% and 85% at 10 and 15 years of followup, respectively.

Radiotherapy and IMRT results are difficult to compare because of the different biochemical relapse criteria. Estimated 10-year biochemical disease-free survival reported in each risk group was 84–70% for low-risk patients, 76%–57% for intermediate-risk Patients, and 55%–41% for high-risk patients. Intermediate- and high-risk results also vary depending on the adjuvant and neoadjuvant treatment with a short- or long-term androgen deprivation [31].

Recent data suggest an equivalent outcome in terms of the BPFS in comparison with high-dose EBRT (HD-EBRT). In a retrospective analysis of modern series, BPFS rates of 85.8%, 80.3%, and 67.8% in men with low-risk, intermediate-risk, and high-risk prostate cancer, respectively, were reported after a mean followup of 9.43 years [32–35].

Donnelly et al. published in 2010 a randomized trial comparing men with localised prostate cancer treated with EBRT versus cryosurgery [17]. Although the sample was quite small (*n* = 244), with a median followup of 100 months, authors cannot rule out inferiority of cryosurgery compared to EBRT at 36 months. Disease progression at 36 months was observed in 23.9% of men in the cryoablation arm and in 23.7% of men in the radiotherapy arm. No differences in overall or disease-specific survival were observed. At 36 months, more patients in the radiotherapy arm had a cancer-positive biopsy (28.9%) compared with patients in the cryoablation arm (7.7%).

There have been no randomised trials comparing brachytherapy with other curative treatment modalities, and outcomes are based on nonrandomised case series. The BPFS after 5 and 10 years has been reported to range from 71% to 93% and from 65% to 85%, respectively, with a median followup ranging from 36 to 120 months [36].

Donnelly et al.'s group [37] also compared series of radical surgery, external beam radiation therapy (EBRT), and brachytherapy with dates of cryosurgery series in medium and high-risk patients. five-year BPFS in medium-risk rates was 37–97% for RP, 26–60% for EBRTs and 66–82% for brachytherapy. In high-risk cancer BPFS decreases to 16–61% in low risk, 19–25% in EBRT and 40–65% in the brachytherapy groups. With this results, authors concluded that the efficacy of cryosurgery appears to be superior to EBRT for moderate- and high-risk patients, and data were comparable in their series to radical prostatectomy and brachytherapy for both medium- and high-risk patients. In this study the definition of biochemical relapse varied between series.

Radiotherapy seems to affect erectile function to a lesser degree than surgery [38]. One-year rates of probability for maintaining erectile function were 0.76 after brachytherapy, 0.60 after brachytherapy + external irradiation, 0.55 after external irradiation, 0.34 after nerve-sparing radical prostatectomy, and 0.25 after standard radical prostatectomy. When studies with more than 2 years of followup were selected (i.e., excluding brachytherapy), the rates became 0.60, 0.52, 0.25, and 0.25, respectively [39]. An increased risk of radiation-induced malignancies of the rectum and bladder following EBRT has been demonstrated [40, 41].

In terms of quality of life, there are several studies comparing surgery with cryotherapy for localized prostate cancer. Men treated with cryotherapy and brachytherapy reported higher urinary symptoms compared to RP [42]. Men treated with brachytherapy have better results in erectile function. Since the moment it was applied, robot assisted prostatectomy has not demonstrated significant advantages in functional outcomes compared to open approaches. A prospective study comparing open, laparoscopic, and robotic radical prostatectomy, brachytherapy, and cryotherapy was recently published [23]. 719 patients from a single institution were evaluated at 1, 3, and 6 months after treatment. Men treated with brachytherapy and cryosurgery were older and had more comorbidities. After this short-term analysis they have found that cryotherapy has a negative impact on urinary function at one month compared with brachytherapy, but this effect disappears at 3 and 6 months; irritative and obstructive symptoms were higher in brachytherapy patients. Cryotherapy patients had worst outcomes in sexual function compared to all other treatments, but baseline function was also lower.

In our series, followup exceeds 10 years in twenty patients and more than a half have up to five years monitoring; still, biochemical recurrence-free survival remains high. The BPFS for low-risk patients is 96.4% and for patients at intermediate risk reaches 91.2% without statistically significant differences between them. For high-risk patients data are favourable (62.2%) and differences are significant. Globally the BPFS is 86.4% without statistically significant differences seen when calculating the BPFS including salvage treatments for biochemical relapse (BPFS 88.1%).

These data are comparable to those published in the literature and using similar criteria for recurrence and outcomes longer than 5 years. Cohen et al. in 2008 [20] reported 370 patients with a median followup 147 ± 33 and results of BPFS of 80%, 74%, and 46% for tumours of low-, intermediate- and high-risk, respectively. In 2010 Donnelly et al. [17] presented 117 patients followed up to 7 years with a global BPFS of 73%. Dhar et al. (CEI Registry) [22] presented 4693 patients with greater than 5 years of followup and a BPFS by 75% prior to the current recurrence criteria (ASTRO = 3 consecutive PSA increases after the posttreatment nadir). Other series as the Bahn's one (7 years of followup) [1], Prepelica et al. (6 years of followup) [43] communicate similar BPFS data but with ASTRO criteria; BPFS for Prepelica was 82% and 92%, 89% and 89% for low, intermediate, and high risk, respectively according to to Bahn.

Our series only included cases of organ-confined disease, except two cases classified as T3a. Indication in the extracapsular cases has been made by the existence of a previous abdominal neoplasm treated with radiotherapy and chemotherapy and life expectancy of less than 5 years. In the literature there are references to series including T3a and T3b cases with freezing of seminal vesicles [44], with acceptable results. In our series, both patients presented PSA kinetics and biochemical relapse criteria confirming metastatic disease. They began ADT treatment with good control of the disease. In both cases, death occurred by causes not related to prostate cancer.

Prostate volume is another factor to take into account. Volumes higher than 45–50 cc. contraindicate cryotherapy as Onik [45] affirms because then areas of the gland could be out of reach of the ice balls diameter and the lethal effect of the cold, and also there would be interference with the pubis. The requirement of greater number of cryoprobes and higher temperature gradients will cause tissue damage to those interposed between two cryoprobes with consequent impairment in the desired effect.

Clinical guidelines of the EAU and AUA refer to the recommended maximum prostate volume, 40 mL and 45 cc respectively, advising the use of hormone therapy to decrease gland volume [17]. In our series the median to the diagnosis was 33 cc, CI 95% 34, 13–40.13. In 19 cases the volume was greater than 45 cc, starting ADT for a period no longer than 6 months. The median volume at the time of treatment was 31 cc with 95% CI 30, 15–35.17. Decrease of volume to less than 45 cc limits occurred in 11 cases.

In 17 cases, three complete freeze-thaw cycles were performed, 8 (7.4%) because of a prostate volume higher than 45 cc, and on 9 occasions for prostates of longitudinal diameter larger than 35 mm, associated also with “pull back” maneuver. No differences were observed in terms of BPFS between two-cycles-treated patients and those treated with a third cycle, mobilization of the cryoprobes independently.

Actually there are no absolute contraindications for the realization of cryosurgery, except the haemorrhagic diathesis and rectal fistulas (inflammatory bowel disease, etc.). Transurethral resection of prostate (TURP) is a relative contraindication; it is associated with a significant higher risk of urethral sloughing because of the difficulty for the coaptation of the urethral warming device. Patients with previous obstructive lower urinary tract symptoms have higher risk of urinary obstruction after treatment. The existence of a significant prostate mid lobe requires a previous treatment before cryosurgery because it will always be out of reach of the ice ball by anatomic location. In our study patients with a mid lobe detected by ultrasound were rejected for treatment. Previous pelvic and urethral surgery that can disturb the anatomy also contraindicated the technique, although according to the literature, not in an absolute way. In these cases, it has been suggested the realization of an urethrocystoscopy in order to assess the integrity of the urethra ensuring the correct placement of the urethral warming catheter. In our series, any patient had prior urethral surgery.

Pathological findings at prostate biopsies after cryoablation include necrosis, fibrosis, hyalinization, microcalcifications, inflammation, stromal haemorrhage, basal cells hyperplasia and transitional and squamous metaplasia, depending on the time relapsed between cryoablation and control biopsy as well as an increase in stroma vascularization. Even some degree of glandular regeneration have been found, which sometimes can reach up to 60% of the total of the material sent to study [46]. This fact, in addition to the conservation of periurethral glands, would justify progressive PSA elevation up to some level of stabilization, but inferior than the criteria for biochemical recurrence.

All these described lesions and a cold-induced effect do not limit the repeat use of cryotherapy. The failures, by tumour recurrence, can be treated with new sessions. No interferences are seen with other therapeutic modalities: hormone treatment or radiation. Moreover, the freezing-induced necrotic areas are surrounded by hyperaemic areas that probably boost the effect of these treatments.

In our series we have treated six cases with a second salvage treatment. It is worth noting that 4 of them are included in the first cases treated. Cases 1, 2, and 12 were made before the rectal Onik manoeuvre was well established. As described in the literature, the highest incidence of complications and the worst results occur in the series published prior to the development of this maneuver. An increase in complications rates has not been appreciated after salvage treatments and all cases are free of biochemical relapse with a maximum followup (132 months) in the first and second cases of the study.

The Cochrane Library in its prostate cryoablation review (Cochrane reviews: Cryotherapy for Localized Prostate Cancer *n* = 1483) reported a rate of incontinence that ranged from 1.3% to 19%, a rate of erectile dysfunction of 47% to 100%, obstruction of 2% to 55%, and fistulae of 0% to 2%. The higher rates of obstruction and incontinence are seen in the older series, using first generation technology, five cryoprobes, and absence of prostato-rectal hydrodistension. The procedure evolution has improved these rates. The 2013 EUA Guidelines describes complication rates of erectile dysfunction in about 80% of patients, tissue sloughing in about 3%, incontinence in 4.4%, pelvic pain in 1.4%, and urinary retention in about 2% (6–11). The development of fistula is usually rare, being <0.2% in modern series. About 5% of all patients require transurethral resection of the prostate (TURP) for infravesical obstruction.

Data of our series reflect a rate of 5.6% incontinence, erectile dysfunction of 98.1% regardless of its presence before treatment, urinary obstruction in 1.9%, and fistulae in 0.9%, with a cancer-specific survival of 98.1%. These findings are consistent with those published in the literature.

## 5. Conclusions

Cryotherapy is an effective treatment and minimally invasive, with low surgical risk, low morbidity, with good results in the long followup in terms of survival, biochemical recurrence, cancer-specific survival and overall survival. It is a valid technique for organ-confined tumours and preferably in low- and intermediate-risk groups. It is a safe alternative for patients with high surgical risk or contraindication for radiotherapy, with a low rate of complications. It can be repeated in case of biochemical relapse after histological confirmation of local recurrence.

When evaluating cryosurgery as a treatment option the main problem is the quality of the data presented to date. The lack of randomized clinical trials and the inexistence of a validated standard definition of failure are the most problematic.

The low rate of complications, with the exception of erectile dysfunction, is a good basis for the future for the election of cryosurgery as the technique of choice for the development of prostatic focal therapy. In fact, although on an experimental basis, it is considered in clinical guidelines.

## Figures and Tables

**Table 1:** Descriptive values.

Patients	
Age (years)	
Median	72.05 (±5,409)
Range	53–81
Associated comorbidity	
Other neoplasms	15 (13.9%)
Cardiovascular pathology	20 (18.5%)
High blood pressure	49 (45.4%)
Diabetes mellitus	21 (19.4%)
Anticoagulant drugs	30 (27.8%)
Prior erectile dysfunction	67 (62%)

**Table 2:** Characteristics and Risk stratification.

Tumor characteristics		
Ng/mL PSA	Median 8.25 (1,818–56,171)	Mean 10.517 95% CI (9,013–12,021)
Prostate volume (cc) at diagnosis	Median 33 (range 11–91)	Mean 37.13 95% CI (34,13–40,13)
Gleason		
5 (3 + 2)	1 (0.9%)	
6 (3 + 3)	54 (50%)	
7 (3 + 4)	19 (17.6%)	
7 (4 + 3)	18 (16.7%)	
8 (4 + 4)	14 (13%)	
9 (5 + 4)	2 (1.9%)	
Stage (adapted according to TNM 2009 classification)		
cT1c	57 (52.8%)	
cT2a	20 (18.5%)	
cT2b	16 (14.8%)	
cT2c	13 (12%)	
cT3a	2 (1.9%)	
Pathological report		
Right side	33 (30.6%)	
Left side	31 (28.7%)	
Bilateral	44 (40.7%)	
RISK (according to D'Amico criteria)		
Low	28 (25.9%)	
Intermediate	34 (31.5%)	
High	46 (42.6)	
Hormone treatment	44 (40.7%)	
Prostate volume at treatment (cc)	Median 31 (range 14–80)	Mean 32,66 95% CI (30.15–35,17)

**Table:** (a)

Risk					Average			
			Censored				95% CI	
	Total number	Events	*N*	Percentage	Estimate	Typical error	Lower limit	Upper limit
Low	28	1	27	96.4%	120,926	3,988	113,090	128,762
Intermediate	34	3	31	91.2%	112,643	6,742	99,429	125,857
High	45	17	28	62.2%	79,554	8,413	6,065	96,044

Gleason								
5 (3 + 2)	1	0	1	100.0%	—	—	—	—
6 (3 + 3)	54	4	50	92.6%	119,321	5,418	108,702	129,939
7 (3 + 4)	19	3	16	84.2%	112,217	10,434	91,767	132,666
7 (4 + 3)	17	5	12	70.6%	90,500	14,373	62,329	118,671
8 (4 + 4)	14	7	7	50.0%	45,093	9,799	25,886	64,299
9 (4 + 5)	2	2	0	0%	—	—	—	—

Global	107	21	86	86.4%	100,254	4,769	90,908	109,600

**Table:** (b)

Pairs comparison							
	Risk	1		2		3	
		Chi-square	Sig.	Chi-square	Sig.	Chi-square	Sig.
Log Rank (Mantel-Cox)	Low			.927	.336	10,337	.001
	Intermediate	.927	.336			6,730	.009
	High	10,337	.001	6,730	.009		

**Table 4:** Salvage cryotherapy.

Case	Risk	Time to relapse (months)	Time tracking (months)	Recurrence	
1	Intermediate	6	126	Not	Exitus non cancer related
2	Low	6	126	Not	
12	Low	15	6	Not	
23	High	6	90	Not	
55	Intermediate	60	48	Not	
78	High	12	15	Not	

**Table 5:** Complication rate.

Complications	
Incontinence	6 (5.6%)
Erectile dysfunction	106 (98.1%)
Obstruction	2 (1.9%)
Urethral sloughing	6 (5.6%)
Haematuria	2 (1.9%)
Pain	12 (11.1%)
Prostato-rectal fistula	1 (0.9%)
